# Percentile model and usual gait speed reference values in Colombian older adults: a secondary analysis of the Survey on Health, Wellbeing, and Aging in Latin America and the Caribbean (SABE)

**DOI:** 10.3389/fragi.2026.1965961

**Published:** 2026-10-19

**Authors:** Philippe Tadger-Viloria, Margareth Lorena Alfonso-Mora, Diana Díaz-Vidal, Aura Cristina Quino-Ávila, John Andrew, Carolina Sandoval-Cuellar, Eliana Monsalve-Jaramillo, Maria Giné-Garriga

**Affiliations:** 1 Universidad de Boyacá, Tunja, Colombia; 2 Vrije Universiteit, Brussels, Belgium; 3 SomaLynk, Brussels, Belgium; 4 Universitat Ramon Llull, School of Psychology, Education and Sport Sciences Blanquerna, Barcelona, Spain; 5 Universidad de San Buenaventura, Cartagena de Indias, Bolívar, Colombia; 6 University of Galway, Galway, Ireland; 7 Universitat Ramon Llull, School of Health Sciences Blanquerna, Barcelona, Spain

**Keywords:** clinical practice, percentile models, physical function, reference values, usual gait speed

## Abstract

**Introduction:**

Gait speed is a simple and clinically relevant indicator of physical function in older adults; however, population-specific reference values are needed for its interpretation. This study aimed to establish reference values and a percentile model for usual gait speed among community-dwelling Colombian older adults and to examine factors associated with slow gait speed.

**Methods:**

Data from 11,945 community-dwelling older adults participating in the Survey on Health, Wellbeing, and Aging in Latin America and the Caribbean (SABE) were analyzed. Slow gait speed was defined as <0.8 m/s. Univariate associations between gait speed and age, sex, height, calf circumference, waist circumference, and urban/rural residence were examined. Reference percentiles for gait speed were modeled using Generalized Additive Models for Location, Scale, and Shape (GAMLSS), incorporating age, sex, height, calf circumference, waist circumference, and body mass index. Model selection was guided by Q statistics and the Generalized Akaike Information Criterion (GAIC).

**Results:**

Gait speed ranged from 0.6 to 1.4 m/s, with a mean of 0.79 m/s and a median of 0.76 m/s. Slow gait speed was more frequent among female than male participants (56% of those with slow gait speed were female). Participants with slow gait speed were older than those without slow gait speed (69.21 vs. 67.53 years). Slow gait speed was significantly associated with older age, female sex, shorter height, lower calf circumference, higher waist circumference, and rural residence. Height accounted for a substantial proportion of the sex-related differences in gait speed, particularly among the oldest participants.

**Discussion:**

This study provides population-specific reference values and percentile estimates for usual gait speed among community-dwelling Colombian older adults. The findings highlight the importance of considering anthropometric characteristics, particularly height, when interpreting sex-related differences in gait speed. These normative values may facilitate the clinical interpretation of gait speed and support assessment and intervention planning in the Colombian older population

## 1 Introduction

Walking is an essential component of daily life, and usual gait speed has been recognized as the “sixth vital sign” of health status (Middleton et al., 2015; Kawai et al., 2021). Gait speed is a simple, inexpensive, and clinically useful measure of physical performance in older adults (Rydwik et al., 2012). It is a reliable and sensitive tool for screening several health conditions and is closely associated with wellbeing, healthy aging, physical frailty, and survival in older populations (Boulifard et al., 2017; Chen et al., 2021; Studenski et al., 2011; VanSwearingen et al., 2011). Additionally, gait speed is a strong predictor of falls, disability, and cardiovascular disease (de la Cámara et al., 2020) and has also been associated with cognitive decline (Cifuentes et al., 2022; Pérez-Sousa et al., 2021).

The 0.8 m/s threshold has established clinical relevance in geriatric assessment and has been widely used to identify impaired physical performance in older adults, including in the assessment of sarcopenia (Cruz-Jentoft et al., 2019) and frailty (Lee et al., 2017). Among available physical performance measures, gait speed is particularly suitable for the routine clinical evaluation of older adults (Guralnik et al., 2000) as it is easy to administer, requires no laboratory equipment, and can be used across healthcare disciplines (Graham et al., 2008). It has also demonstrated high interrater and test–retest reliability (Studenski et al., 2011). Taken together, these characteristics support the use of gait speed as a practical and informative measure of physical function in both clinical and community settings.

Gait speed has been shown to vary with age, sex, height, and body-mass index (BMI). Specifically, gait speed is typically faster in younger older adults (Pérez-Sousa et al., 2021), and even though age-related decline is observed in both sexes, female individuals generally exhibit slower gait speeds. Height is also a well-documented determinant of gait speed, with taller individuals generally walking faster due to longer stride lengths and lower cadence for a given pace. This biomechanical relationship between leg length (as a proxy for height) and gait performance has been consistently reported across populations (Kasović et al., 2021; Mikos et al., 2018). BMI further contributes to gait variation, with higher values often linked to slower gait due to increased energy demands and altered biomechanics (Kawai et al., 2019). Usual gait speed in older adults reported by previous studies is around 0.94–1.34 m/s, with slower speed than 0.8 m/s being linked to a higher risk for falls (Kasović et al., 2021).

Several studies conducted with Colombian older adults have shown findings similar to other worldwide studies, reporting significant associations between a slower gait speed and being older (with the highest difference after 80 years old), being female, and obesity (Ramírez-Vélez et al., 2019; Cifuentes et al., 2022). In another study conducted in Colombian older adults, sarcopenic status was also associated with poor gait speed and functional dependence (Pérez-Sousa et al., 2019). No previous study among Colombian older adults had reported reference values of usual gait speed, which could be of interest to targeting individuals for primary prevention and estimating the proportion of fast or slow values for gait speed in community-dwelling Colombians aged over 60 years old.

One study used the Survey on Health, Wellbeing, and Aging in Latin America and the Caribbean (SABE) to establish reference values of the short physical performance battery (SPPB) for community-dwelling older Colombian adults, including gait speed (as one of the three tests included in the SPPB) (Ramírez-Vélez et al., 2020); however, they did not explore the normative values of usual gait speed in the Colombian population. Additionally, the current study offers a web application that allows the use of these cut-off points to evaluate the usual gait speed percentile points in which the patient is located, which is useful for screening the older adult population. Thus, the objective of this study was to provide a percentile model and establish usual gait speed reference values for community-dwelling Colombian older adults using a secondary analysis of the SABE.

## 2 Methods

### 2.1 Design and participants

A secondary analysis was conducted on the cross-sectional 2015 SABE survey (Albala et al., 2005) on health and aging in Colombia. Elderly subjects (60 years or older) from all over Colombia, male and female, were evaluated physically and cognitively. A probabilistic clustered, stratified/multistage sample was used from urban and rural areas. All participants signed their informed consent prior to data collection. The ethical aspects of the study were approved by the University of Caldas (ID protocol CBCS-021-14) and the University of Valle (ID protocol 09-014 and O11-015). This secondary analysis was approved by Universidad de Boyacá (04-Jun-2021). The technical details of the SABE survey are published in Gómez et al. (2016).

From the initial 23,510 participants, a complete-case analysis was performed on the response variable and predictors. Observations that were missing gait speed, age, height, weight, BMI, calf circumference, waist, or sex were removed. Implausible values were excluded: height/weight = 0; weight > 175 kg; BMI < 12 or > 150; calf circumference < 25 or > 45; gait speeds ≤ 0.4 m/s (6.58%). Participants > 90 years (125, 0.53%) were excluded due to low representation. Only those with gait speeds between 0.6 and 1.4 m/s were retained, excluding 5,144 (21.9%) strictly between 0.4 and 0.6 m/s, and 81 (0.34%) greater than 1.4 m/s. The latest filter aimed to optimize model fit and meet quantile regression assumptions. The final sample comprised 11,945 participants (49.2% of the original sample).

### 2.2 Study variables

A structured interview was administered to obtain socio-demographic data (age, sex, and rural or urban zone). Anthropometric measures were collected: height was measured with SECA 213®, Hamburg, Germany to the nearest 0.1 cm, and weight with a Kendall graduated platform scale with a precision of 0.1 kg; both were measured with the subject wearing light indoor clothing. BMI was calculated using the formula “BMI = weight (kg)/height (m^2^)”. Calf (CC), arm (CA), and waist (WC) circumferences were measured to the nearest 0.1 cm in a standing position using a non-elastic tape. CC is the circumference of the widest section of the distance between the ankle and the knee (calf area). WC includes the circumference in the abdominal area, at an intermediate level between the last costal arch and the iliac crest, at the narrowest position of the abdomen. Trained staff assessed usual gait speed using standardized procedures for instructions, positioning, and scoring (Ramírez-Vélez et al., 2020). The patient was asked to walk 3 m twice at a usual pace, and the best outcome was registered. All data were taken from the SABE technical report (Ramírez-Vélez et al., 2020).

### 2.3 Statistical methods

Generalized Additive Models for Location Scale and Shape (GAMLSS) was used to fit the gait speed data. This implementation allowed modeling up to four parameters in a distribution family: the mean ( μ ), variance ( σ ), skewness ( ν ), and kurtosis ( τ ) of the distribution. Different covariates were fitted individually for each GAMLSS parameter.

The GAMLSS model (also referred as quantile/centile regression) distribution considered were BCT (Box–Cox t), BCCG (Box–Cox Cole and Green), BCTo (Box–Cox t original), BCPE (Box–Cox power exponential), and BCPEo (Box–Cox power exponential original). The variables that were considered for the adjustment of the quantile model were age, sex, height, weight, BMI, and calf circumference. These variables were incorporated into the quantile model allowing a non-parametric adjustment, using Box–Cox transformation and polynomials splines.

The selection of the best quantile model was based on the Q statistics and Generalized Akaike Information Criterion (GAIC) (Van Buuren and Fredriks, 2001). GAIC can be used where two or more models have similar diagnostic performance. The best full quantile model (with all the variables) was used to fit the predictive quantile curves, and the best quantile models using only age as a covariate and another with height as a covariate were used to present the gait speed predictive values by sex.

A model was fitted separately for male and female individuals. The best GAMLSS model was selected for multivariate (all previously mentioned covariates) and for univariate context (only age and sex). The most parsimonious model (univariate) was used to develop the web app.

## 3 Results

The slowest usual gait speed found in the study sample was 0.6 m/s, and the fastest was 1.4 m/s, with the mean and median being 0.79 m/s and 0.76 m/s, respectively. As seen in Table 1, a usual gait speed of less than 0.8 m/s was considered slow and, above this value, not-slow gait speed. Most female individuals (56%) had slower gait speed than male individuals (44%), while most male individuals dominated the highest values of gait speed (61%) compared to female individuals (39%). Those with slower gait speeds had a mean age of 69.21 years as compared to 67.53 years for those with faster gait speeds. Slow gait speed was associated with older age, being female, having smaller calf circumference and greater waist circumference, being shorter, and living in a rural area. Table 2 shows the odds ratio and the corresponding 95% confidence interval for all covariates.

**TABLE 1:** Gait speed and associated factors.

Variable	High 0.80 m/s, N = 4,716^1^	Less 0.80 m/s N = 7,229^1^	*p*-value^2^
Age	67.53 (5.84)	69.21 (6.54)	< 0.001
Gender	​	​	< 0.001
Female	1,852 (39%)	4,014 (56%)	​
Male	2,864 (61%)	3,215 (44%)	​
Height	1.60 (0.09)	1.57 (0.09)	< 0.001
Weight	79.65 (23.61)	77.49 (23.07)	< 0.001
Circ_calf	34.75 (3.30)	34.48 (3.43)	< 0.001
Circ_arm	28.45 (4.10)	28.47 (4.37)	0.9
Waist	91.86 (10.59)	92.34 (10.83)	0.021
Living area	​	​	0.054
Municipal	3,458 (73%)	5,184 (72%)	​
Rural	1,258 (27%)	2,045 (28%)	​

1 Mean (SD); n (%).

2 Wilcoxon rank sum test; Pearson’s Chi-squared test.

**TABLE 2:** Logistic multivariate model for slow/not-slow gait speed. Slow gait speed < 0.8 is considered a risky condition, and not-slow gait speed ≥0.8 could be considered a protective biomarker.

Characteristic	OR^1^	95% CI^1^	*p*-value
Age	1.05	1.04, 1.05	< 0.001
Gender			
Female	—	—	​
Male	0.54	0.49, 0.60	< 0.001
Height	0.31	0.17, 0.56	< 0.001
Weight	1.00	1.00, 1.00	0.015
Circ_calf	0.97	0.95, 0.98	< 0.001
Circ_arm	1.00	0.99, 1.02	0.6
Waist	1.02	1.01, 1.02	< 0.001
Living area			
Municipal	—	—	​
Rural	1.17	1.07, 1.27	< 0.001

1 OR, odds ratio; CI, confidence interval.

Although calf circumference was significantly associated with slow gait speed in the logistic regression analysis using the 0.8 m/s threshold, it did not significantly contribute to the GAMLSS percentile model. Therefore, calf circumference was not retained to construct the percentile curves.

### 3.1 Full fitted model

We fitted a model with the variables that had been reported as affecting gait speed among male and female individuals. We included age, height, weight, and calf circumference as covariates. We then used *p*-values and plots to remove the covariates that were not significant.

Gait speed has been associated with sex, age, and height. It seemed to be constant over different CC and weight among male and female individuals. CC and weight were not significant (*p*-value 0.05), and hence they were dropped from the model. Thus, a model with age and height was fitted for both male and female individuals (Data available upon request).

Since covariates may sometimes not be linearly associated with the response variable, some transformations were investigated to see whether there was a need for transformation for age and height. The best family was BCPEo for the male and female groups. The suggested power for age for male individuals was 0.30, and for female individuals, it was 0.66. The power for height was too small to be used and hence was not transformed (Data available upon request).

### 3.2 Parameter estimates

Data available upon request shows the parameter estimates for the model with age and height for male. Age and height significantly affected gait speed (*p*-value 0.05) among male individuals. As subjects age by 1 year, gait speed decreases by 0.005 m/s equivalently to 0.1 m/s (10 cm/s). Height had a positive association with gait speed, as the latter increased linearly with an increase in height by 0.15 m/s for every 1-m increase in height equivalent to an increase in gait speed of 0.015 m/s for every 0.1 m (10 cm) in height.

Since the usual gait speed for male individuals seems to associated with both age and height while that of female individuals is only associated with age, we fitted and compared a model with age and height to a model with age only among male and female individuals.

In the subsequent sections, we refer to Model 1 as the model with age only as a covariate and Model 2 as the model with age and height as the covariates.

### 3.3 Model comparison

We compared Models 1 and 2 using AIC (Akaike Information Criteria). Table 3 shows the AIC for the two models for male individuals.

**TABLE 3:** AIC for the fitted models for male and female.

Model	df	AIC
b2.Male.linear	14.31	−7430
b2.Male.age	10.35	−7419
b2.Female.age	9.7	−9958
b2.Female.linear	13.79	−9956

From Table 3, the model with both age and height (b2. Male.linear-b2.Female.linear) seems to be the best model for male and female individuals.

#### 3.3.1 Prediction of gait speed

For prediction purposes, we used Model 1 (with age only as a covariate) (Data available upon request). This was to have coherence between male and female populations, since only age was the only covariate associated with gait speed for both male and female individuals.

We predicted gait speed for different age groups using Model 1 (with age only as a covariate) for both male and female individuals. As shown in Table 4, gait speed (5–95 percentiles) decreased with age in both male and female populations.

**TABLE 4:** Predicted gait speed for male and female individuals in different age groups.

Age-male	N	P5	P10	P25	P50	P75	P90	P95
60–70	3709	0.63	0.65	0.71	0.80	0.93	1.05	1.12
71–80	1935	0.62	0.64	0.68	0.76	0.87	0.99	1.07
81–90	435	0.62	0.63	0.66	0.72	0.82	0.92	0.99
Age-female								
60–70	3990	0.62	0.63	0.66	0.73	0.84	0.96	1.03
71–80	1600	0.61	0.62	0.65	0.71	0.80	0.90	0.96
81–90	276	0.61	0.62	0.64	0.68	0.76	0.85	0.91

#### 3.3.2 Centile curves for model 1

The fitted centiles for Model 1 for male and female individuals are shown in Figure 1 (fitted centiles using model 1 for male and female individuals).

**FIGURE 1:**
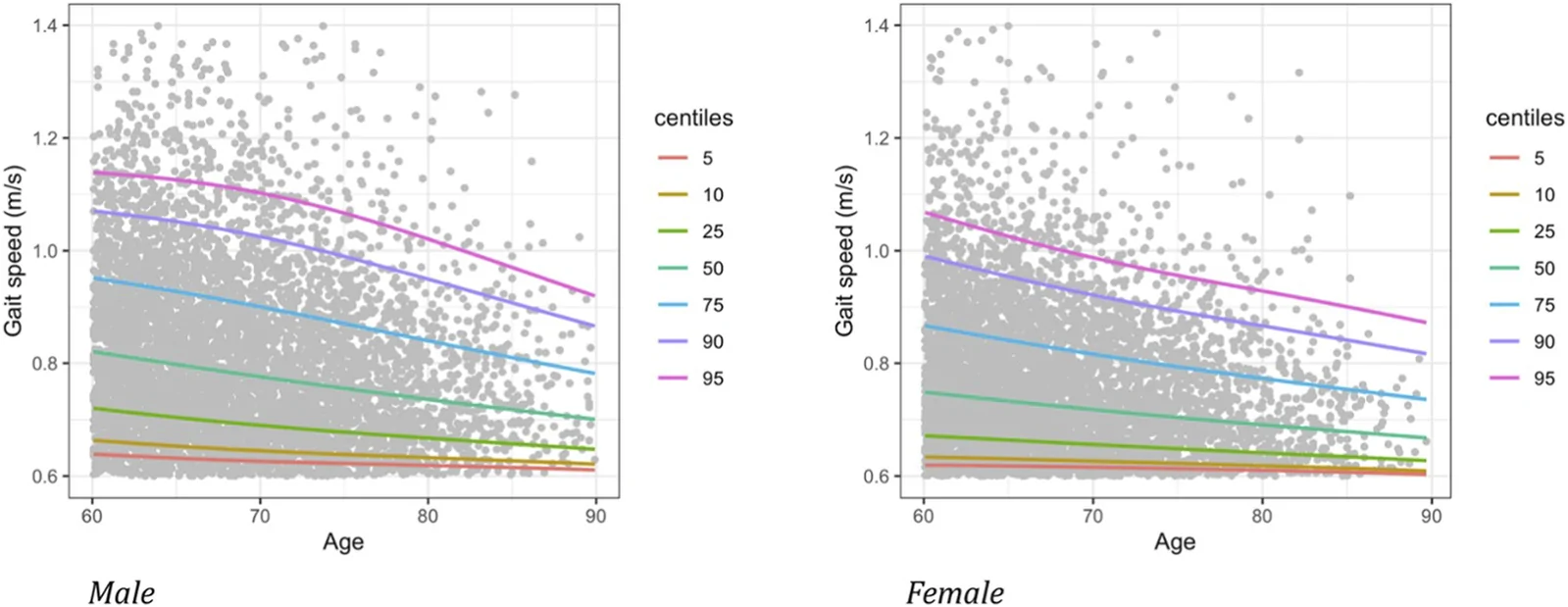
Percentile Curves for Gait Speed in Adults Aged 60 Years and Older: Stratification by Sex.

### 3.4 Model 1 for different heights for male and female individuals

Since height was not significant among female individuals but significant among male individuals, we accounted for it by fitting Model 1 to different groups of people with different heights..

**FIGURE 2:**
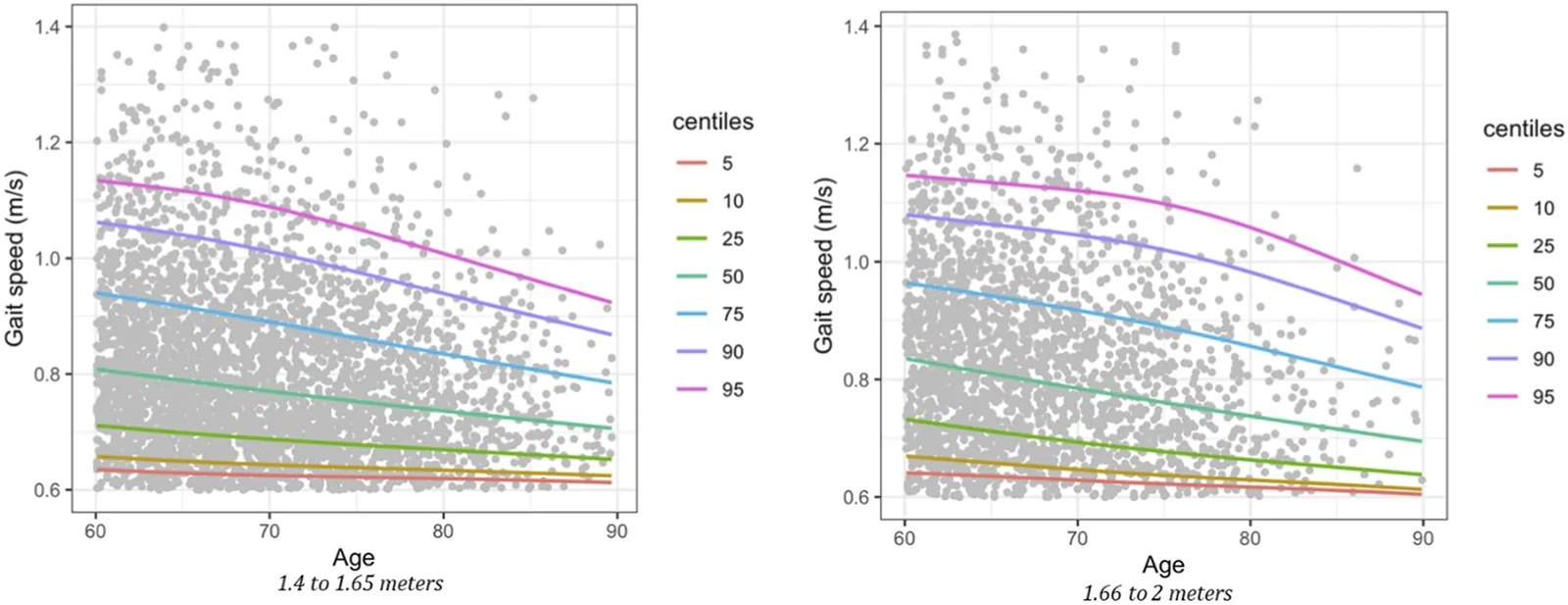
Percentile Curves for Gait Speed in Male Adults Aged 60 Years and Older: Stratification by Height.

We divided the data based on the height of the participants into two groups.

1. Male: 1.40–1.65 m (group 1) and 1.66–2.00 m (group 2).
2. Female: 1.40–1.49 m (group 1) and 1.50–2.00 m (group 2).
3. Group 1 for male individuals had 3,717 observations, and 2,362 observations for group 2. Group 1 for female individuals had 2238 observations, and 3628 observations for group 2. The division was done by balancing the number of observations between groups 1 and 2; thus, the cut points for height among male and female individuals were chosen arbitrarily to achieve a balanced sample between groups. Model 1 was then fitted in each group. Figure 2 shows the fitted centiles for the two groups for male and female individuals (fitted centiles using Model 1 for male: height).

Figure 3 shows the contour plot for female individuals for different combinations of height and age (fitted centiles using Model 1 for female: height).

**FIGURE 3:**
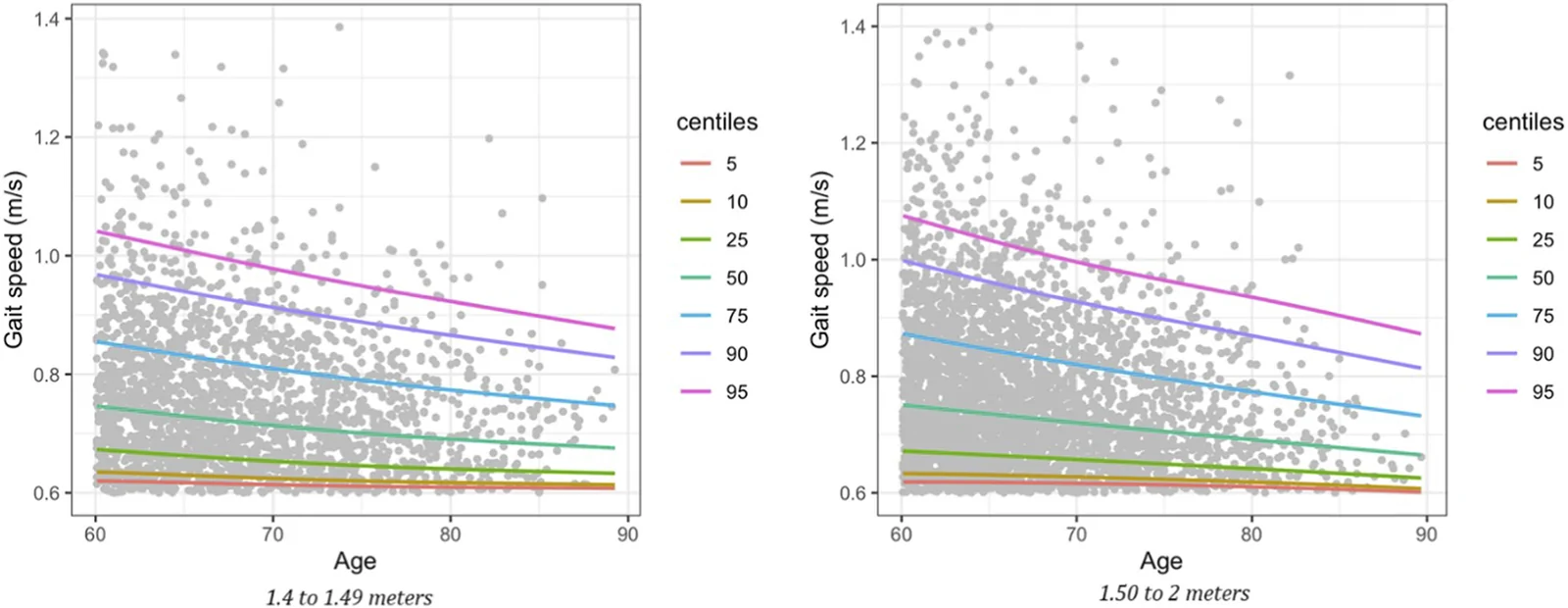
Percentile Curves for Gait Speed in Female Adults Aged 60 Years and Older: Stratification by Height.

## 4 Discussion

This secondary analysis offers a percentile model and usual gait speed reference values in a large sample of community-dwelling Colombian older adults. The model was constructed based on age and sex, and it was further adjusted for participants’ height parameters after excluding other irrelevant covariates, such as calf circumference and weight, which could potentially affect the model.

An additional strength of this study is the use of GAMLSS, which provides greater flexibility than conventional linear regression by allowing different parameters of the gait speed distribution to be modeled and by accommodating potential non-linear relationships between gait speed and relevant covariates.

The current percentile model encompasses a range from the 5th to the 95th percentile. Notably, for men aged 60–70, the 50th percentile shows a walking speed of 0.80 m/s, while for women it was 0.73 m/s. In both sexes, there was a decline in usual gait speed with each passing decade. However, this decline was more pronounced in men than women—a trend consistent with findings from other studies. Male individuals decreased 5% each decade (P50) in comparison to female individuals, who decreased 3%.

Female individuals had a 9% slower gait speed than male individuals (male: 0.80 m/s; female: 0.73 m/s). This decline has been reported by other studies, however, with 21.4% and 27.9% in male and female individuals, respectively. Kasović et al. (2021) and Bergland and Strand (2019) reported that the decrease in usual gait speed was similar in both sexes until 80 years old, but after then it decreased significantly in women.

For our study, the mean age of older adults with a slower gait speed was 69.21 years old, and this covariate has an inverse relationship with walking speed. Some studies reported that the age at which gait speed begins to decrease is 60 years old (Kasović et al., 2021; Pua et al., 2022; Sialino et al., 2021). Our results showed that slower gait speed was associated with waist circumference (CI: 1.01–1.02; *p* < 0.001), coinciding with findings of Mikos et al. (2018) (r = - 0,28 years −0,21, *p* < 0.001).

Regarding height, our study found that gait speed was faster when older adults were taller. While gait speed can be normalized by height to account for anthropometric differences, we opted to include height as a covariate in the GAMLSS models. This allowed us to directly assess its independent effect on the distributional characteristics of gait speed while preserving interpretability in clinical and public health contexts. Additionally, by stratifying the sample by height categories and presenting percentile curves accordingly, we accounted for height-related variability in a way that aligns with how gait speed is typically used in practice. Although height was not a statistically significant predictor of gait speed among female participants in the multivariate model, this may be due to the narrower and more concentrated distribution of height in this subgroup. Compared to male participants, female participants showed less variability in height, which could limit the explanatory power of this variable in the model. Stratified analyses by height groups, however, did reveal meaningful differences in gait speed within both sexes, supporting the relevance of height as a modifying factor.

The reference values of mean height for European populations are 1.76 m in male individuals and 1.63 m in female individuals, which differs from Colombians, who are shorter than Europeans (1.71 m in male and 1.58 m in female individuals). According to the regression model employed by Mikos et al. (2018), increased height is linked to faster walking speed, with a 10 cm increase in height explaining a 5 cm extension in stride length and a reduction in cadence by 5.6 steps/min. The direct relationship between height and walking speed justifies the need for research in various ethnic groups so as not to compare normative values of such variables with non-comparable populations (Yoda et al., 2021).

Beyond its value as a descriptive measure of physical performance, gait speed is increasingly recognized as an indicator of future functional limitation and disability. Recent longitudinal evidence supports this clinical relevance. In a 9-year follow-up study of community-dwelling older adults, slow gait speed was associated with subsequent dependence in basic activities of daily living and thus independently predicts functional dependence (Marincolo et al., 2024). More recently, an analysis of the English Longitudinal Study of Ageing found that low physical performance, defined as gait speed ≤ 0.8 m/s, was more effective than the overall constructs of frailty and sarcopenia in identifying the risk of incident disability in both basic and instrumental activities of daily living (De Souza et al., 2025). These findings are particularly relevant to our study, as they support the clinical significance of the 0.8 m/s threshold used to characterize slow gait speed and reinforce the potential utility of population-specific reference values for identifying older adults at increased risk of functional decline.

Gait speed should also be considered a dynamic marker that reflects changes in health and functioning over time rather than solely a cross-sectional measure of mobility. Recent longitudinal studies have shown that gait speed progressively declines with aging and that its trajectory may provide additional prognostic information. Pollak et al. (2024), for example, reported age-related declines in gait speed among community-dwelling older adults and showed that social factors, particularly tangible social support, were associated with a slower rate of decline. In addition, Perera et al. (2024) demonstrated that repeated gait speed measurements were strongly associated with mortality risk, supporting the value of the longitudinal monitoring of gait speed in older populations. Taken together, this evidence strengthens the relevance of the age- and sex-specific percentile curves presented in our study, which may provide a useful reference for detecting individuals whose gait performance is below that expected for their demographic characteristics and for monitoring changes that could precede clinically meaningful functional deterioration.

This study enables us to discern frailty indicators, fall markers, incident disability, and mortality risks within the older adult population. This variable, due to its cost-effectiveness and ease of implementation, proves highly advantageous, especially in community or primary care settings. The reference values and centile curves presented here are intended to offer a practical and interpretable tool for clinicians and researchers who work with older adults in Colombia. Although height has a recognized effect on gait speed—particularly in men—we accounted for this by incorporating height as a covariate in our models and presenting stratified analyses by height groups. While normalizing gait speed by height is a valid analytical approach, it is not commonly used in clinical practice. Our percentile-based reference values, stratified by age and sex, are therefore more readily applicable in real-world settings where gait speed is measured routinely. This should enhance the clinical utility of our findings, particularly for early identification of functional decline and frailty in community-dwelling older adults.

Turning to the study’s limitations, while numerous variables can be utilized as predictors to control for confounding factors, their graphical representation remains unfeasible as each variable necessitates its own dimension. We explored two visualization alternatives (contour plots and the division of the population into age and height groups) in an effort to retain two crucial variables (age and height, in addition to sex). We consider this limitation more a byproduct of current statistical modeling and software capabilities than a unique constraint inherent to our study. A further limitation is that the reference values were derived from the 2015 SABE Colombia survey. Potential cohort and generational changes in health, lifestyle, anthropometric characteristics, and physical functioning may influence gait speed distributions over time. Therefore, these reference values should be periodically reassessed and validated using more recent cohorts of Colombian older adults.

## 5 Conclusion

We present clinically valuable and easy-to-use reference values for usual gait speed in community-dwelling Colombian older adults. Our findings show that gait speed decreases with age and differs between male and female populations, with height explaining an important part of the observed sex-related differences. The age- and sex-specific percentile values provide a population-specific framework for interpreting gait performance in Colombian older adults. These reference values may facilitate the identification of individuals with lower-than-expected gait speed and help identify older adults potentially at risk of functional decline and frailty in clinical and community settings. Given that gait speed is a simple, inexpensive, and easily implemented measure of physical performance, these findings may support its wider use in routine assessment and intervention planning. Future longitudinal studies should examine the ability of these percentile values to predict relevant health outcomes, including functional limitations, falls, disability, and mortality.

## Data Availability

The authors are not authorized to redistribute the original data, so they cannot share the dataset publicly. Anonymized microdata are available from the Ministry of Health and Social Protection upon registration and acceptance of its terms of use, through its Population Studies and Surveys portal: https://ghdx.healthdata.org/record/colombia-health-well-being-and-aging-survey-2015.
